# Short-loop engineering strategy for enhancing enzyme thermal stability

**DOI:** 10.1016/j.isci.2025.112202

**Published:** 2025-03-11

**Authors:** Wenlong Zhu, Yiheng Liu, Hui Cao, Luo Liu, Tianwei Tan

**Affiliations:** 1 National Energy R&D Center for Biorefinery, Beijing University of Chemical Technology, No. 15 North 3rd Ring East Road, Beijing 100029, P.R. China

**Keywords:** natural sciences, biological sciences, biotechnology, enzyme engineering

## Abstract

Highly flexible regions were targeted for successful modification to enhance enzyme stability. However, this approach could not cover all key sites. Residues in certain rigid regions are also crucial for protein stability. This study proposed a short-loop engineering strategy that explores rigid “sensitive residues” in short-loop regions and mutated them to hydrophobic residues with large side chains to fill the cavities, thereby improving enzyme thermal stability. This strategy identified sensitive residues in the short-loop regions of three enzymes: lactate dehydrogenase from *Pediococcus pentosaceus*, urate oxidase from *Aspergillus flavus*, and D-lactate dehydrogenase from *Klebsiella pneumoniae*. Under the guidance of the short-loop engineering strategy, the half-life periods of these three enzymes were 9.5, 3.11, and 1.43 times higher than wild type, respectively. We also proposed a standard procedure for this strategy and developed a visualization plugin, offering new insights into enzyme stability modification.

## Highlights

• Mining rigid “sensitive residues” on short loops to enhance stability
• Short-loop engineering strategy was successfully applied to three kinds of enzymes
• A standard procedure and visualization plugin were proposed

## Graphical abstract

**Figure:**
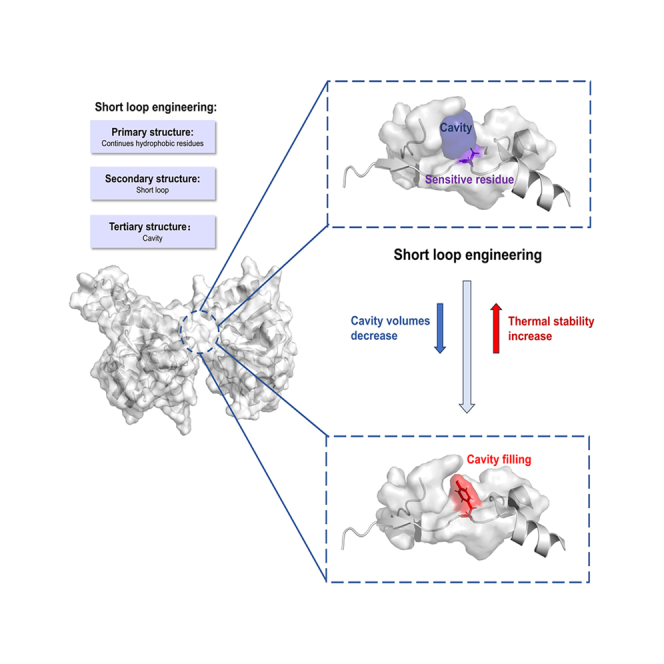

## Introduction

Enzymes, as highly efficient catalysts, play a crucial role in biocatalysis and synthetic biology.^1^ The thermal stability of enzymes is vital for both human health and industrial applications, attracting considerable international attention.^2^ Enhancing thermal stability can improve the reuse rate and half-life of industrial enzymes, significantly reducing operational costs. Additionally, operating at higher temperatures can accelerate enzymatic reaction rates, increase substrate solubility, and lower the risk of microbial contamination.^3^^,^^4^

Enzyme thermal stability can be categorized into thermodynamic and kinetic stability, both of which are influenced by factors such as hydrophobic interactions, hydrogen bonds, salt bridges, and disulfide bonds.^5^^,^^6^^,^^7^^,^^8^ To address these characteristics, various molecular modification strategies have been developed to improve enzyme thermal stability. Among these, directed evolution, often coupled with high-throughput screening, is a widely used method.^9^^,^^10^ Another common approach is the B-factor strategy, which rigidifies flexible regions to reduce the “wobble” of flexible structural domains, thereby improving protein stability.^11^^,^^12^

In recent years, several computational tools for protein stability, such as Fireprot, PROSS, and GRAPE, have been developed. These tools employ un/folding free energy calculations, combined with consensus analysis, to conduct virtual screening and predict the effects of mutations on stability.^13^^,^^14^^,^^15^ Other techniques, such as surface charge reorganization and the introduction of disulfide bonds, have also been utilized to enhance enzyme thermal stability.^16^^,^^17^ More recently, machine learning techniques, including deep learning and deep neural networks, have gained prominence in predicting protein evolution and improving stability.^18^^,^^19^^,^^20^

The loop regions constitute an important secondary structure of proteins, and deeper investigations have revealed that these regions play a crucial role in protein folding, structural maintenance, and catalytic function.^21^ Consequently, loop engineering has emerged as a significant tool for molecular modifications of proteins, particularly in enhancing enzyme stability, activity, and selectivity.^22^ In efforts to improve enzyme thermal stability using loop engineering, techniques such as residue mutation, fragment replacement, and loop deletion are commonly employed.^23^ Additionally, rigidifying loop regions can enhance substrate channel stability, thereby improving catalytic activity.^24^ Some enzymes exhibit broad substrate specificity, where loop regions near active sites play a key role in substrate recognition and binding, thus influencing substrate specificity. Modifying these loop regions to alter substrate specificity has, therefore, become a focal point of research.^25^ As the understanding of proteins and loop regions deepens, loop engineering is playing an increasingly important role in protein molecular modifications.

At high temperatures, proteins are unable to maintain their native conformation, leading to structural loosening and hindering catalytic processes. Therefore, strategies aimed at enhancing stability essentially improve a protein’s ability to retain its native conformation under elevated temperatures. Approaches such as the B-factor method for rigidifying flexible regions, computational designs to increase hydrogen bond numbers, and introducing new disulfide bonds fundamentally restrict proteins to maintain stability under high-temperature conditions. Thermophilic proteins typically contain a higher proportion of hydrophobic and charged residues, clustering hydrophobic residues in the protein core to minimize structural voids.^8^ Consequently, strategies such as cavity filling have been developed.^26^ The loop region serves as a critical junction linking the remaining secondary structures of the protein, influencing interactions between various structural domains.^24^ This study introduces a strategy of short-loop engineering, aiming to identify “sensitive residues” within the short loop and enhance enzyme thermal stability by filling cavities with large side-chain residues. The exploration and filling of cavities in short loops essentially form a strategy for enhancing protein stability through structural constraints. This strategy has been validated on three enzymes— lactate dehydrogenase from *Pediococcus pentosaceus* (PpLDH), urate oxidase (UOX), and D-lactate dehydrogenase (LDHD)—demonstrating its broad applicability. The study proposes a standardized protocol for implementing this strategy and presents a visualization plugin for the rapid identification of sensitive residues in short-loop regions. This approach distinguishes itself from traditional modifications targeting flexible regions and demonstrates universality, offering a specific perspective for enhancing enzyme thermal stability.

## Results and discussion

### Explore the “critical sites” in short-loop engineering

Short loops typically consist of only a few residues, and identifying which of these residues are critical for thermal stability is essential. In this study, we selected PpLDH. We employed a virtual saturation screening strategy based on un/folding free energy calculations to identify “critical sites” within short-loop regions. A six-residue loop in PpLDH, comprising Asn96-Val97-Pro98-Ala99-Tyr100-Ser101, was identified. Using FoldX, we calculated the un/folding free energy (ΔΔG) to perform virtual screening. Virtual saturation mutagenesis, detailed in Table S1, revealed that mutations at Asn96, Val97, Pro98, Tyr100, and Ser101 generally destabilized the protein, as most ΔΔG values were greater than zero. In contrast, Ala99 exhibited a different pattern, with 14 mutant variants showing ΔΔG values less than zero, indicating that Ala99 is the most promising site for enhancing stability within this short-loop region.

**Figure 1:**
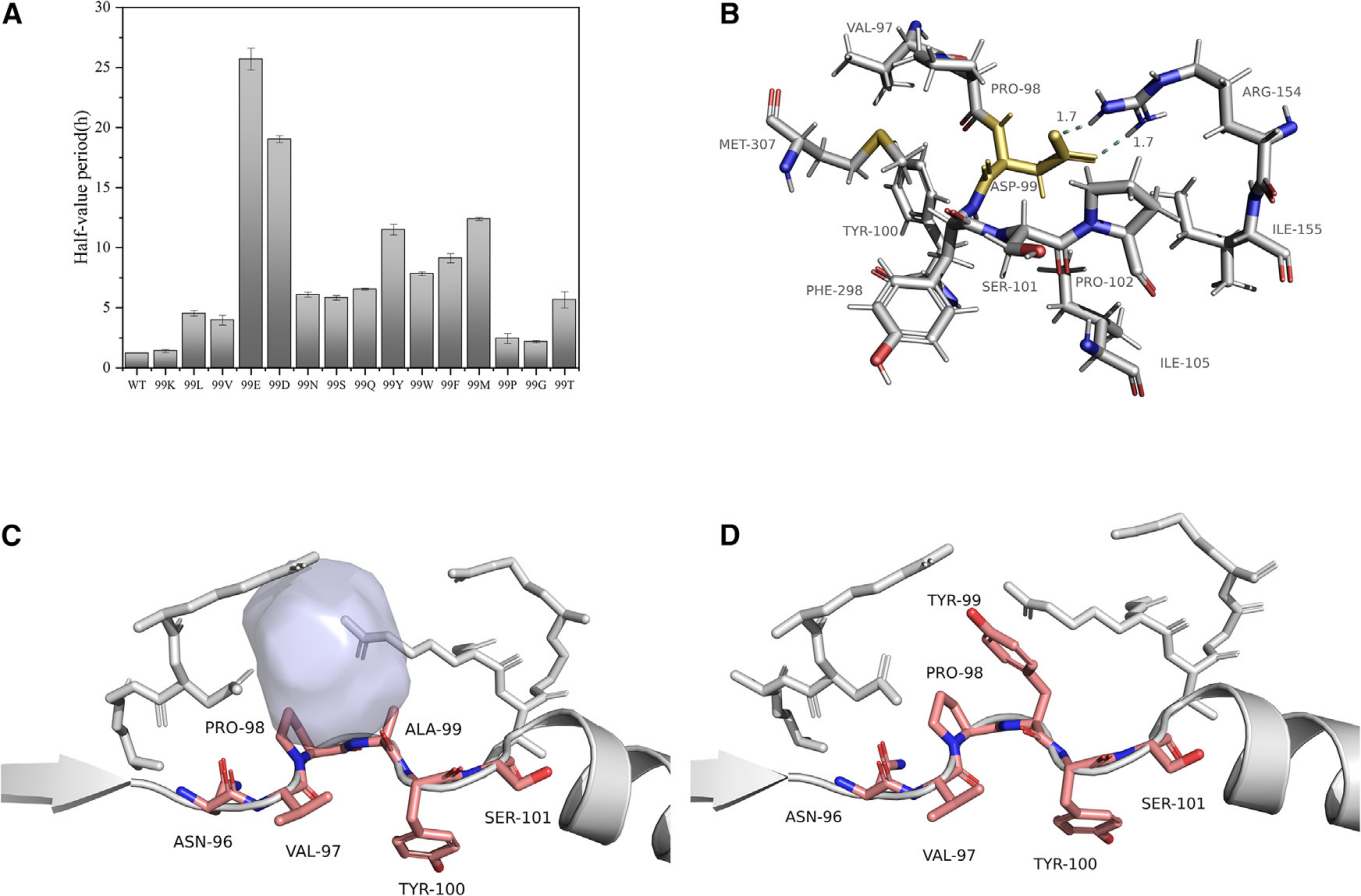
The exploration of “critical sites” in short-loop engineering (A) Half-life period at 45°C of mutants at position 99 following saturation mutagenesis. Values are shown as mean ± SD (*n* = 3 replicates). (B) A99D enhanced overall protein stability through the formation of a new hydrogen bond with ARG154. (C) Wild-type PpLDH showed a noticeable cavity at position Ala99 (marked with a solid blue sphere). (D) The cavity at position 99 was filled upon mutation of Ala to Tyr.

Consequently, we constructed a saturation mutagenesis library for Ala99 and performed expression validation. Nearly all mutations positively affected stability. As shown in Figure 1A, mutations to Glu (99E) and Asp (99D) produced the most significant improvements. Both Glu and Asp are acidic amino acids containing carboxyl groups. Molecular modeling and dynamics simulations of 99E and 99D, as depicted in Figure 1B, revealed that the mutations introduced new hydrogen bonds with Arg154, a key factor contributing to enhanced stability.

Furthermore, mutations to hydrophobic residues with large side chains, such as Met (M), Tyr (Y), Phe (F), and Trp (W), also significantly improved stability. Molecular dynamics simulations and structural analysis revealed a continuous hydrophobic segment within the short-loop region (Asn96-Val97-Pro98-Ala99-Tyr100-Ser101), as illustrated in Figure 1C, with Ala99 located within this segment. Due to the small size of the alanine side chain, which consists solely of a methyl group, Ala99 represented a “vulnerable” position within the short-loop region.

Analysis of the wild-type protein structure revealed that Ala99 indeed creates a noticeable cavity, with a calculated volume of 265 Å^3^. However, mutating Ala to Tyr filled this cavity with the phenyl ring of Tyr, reducing the cavity volume to less than 48 Å^3^ as shown in Figure 1D, without introducing new hydrogen bonds or salt bridges. Hydrophobic residues such as Phe, Trp, Tyr, and Met, known for their strong hydrophobic interactions, filled the “cavity” created by Ala99, thereby enhancing stability. This mechanism differs from traditional methods that rely on creating new hydrogen bonds, salt bridges, or disulfide bonds to improve protein stability.

### Characteristics of short-loop engineering

The following are the characteristics of short-loop engineering.

Cavities in the short-loop region: based on the analysis of experimental results, a 265 Å^3^ cavity was observed at the position of Ala99. When mutations were introduced with large side-chain residues such as Phe, Tyr, or Trp, this void was filled, leading to enhanced stability. Therefore, it can be concluded that residues on the short loop affect protein stability by creating voids, and stability is improved when these voids are filled.

Hydrophobic interactions enhance protein stability: the short-loop region, consisting of Asn96, Val97, Pro98, Ala99, Tyr100, and Ser101, features residues with strong hydrophobic interactions surrounding Ala99, such as Val, Pro, and Tyr. When Ala99 was substituted with residues like Tyr, Trp, or Phe, which have larger side chains and stronger hydrophobic interactions, the hydrophobic interactions in this short-loop region were further enhanced. It is well established that proteins achieve their correct spatial structure through self-folding, a process critical to their proper functioning. Hydrophobic interactions are a key driving force in protein folding and maintaining the correct three-dimensional structure. Mutation at the cavity site significantly enhances the hydrophobic interactions of this segment, providing more rigid support for the entire protein scaffold. This highlights the characteristic benefits of short-loop engineering.

**Figure 2:**
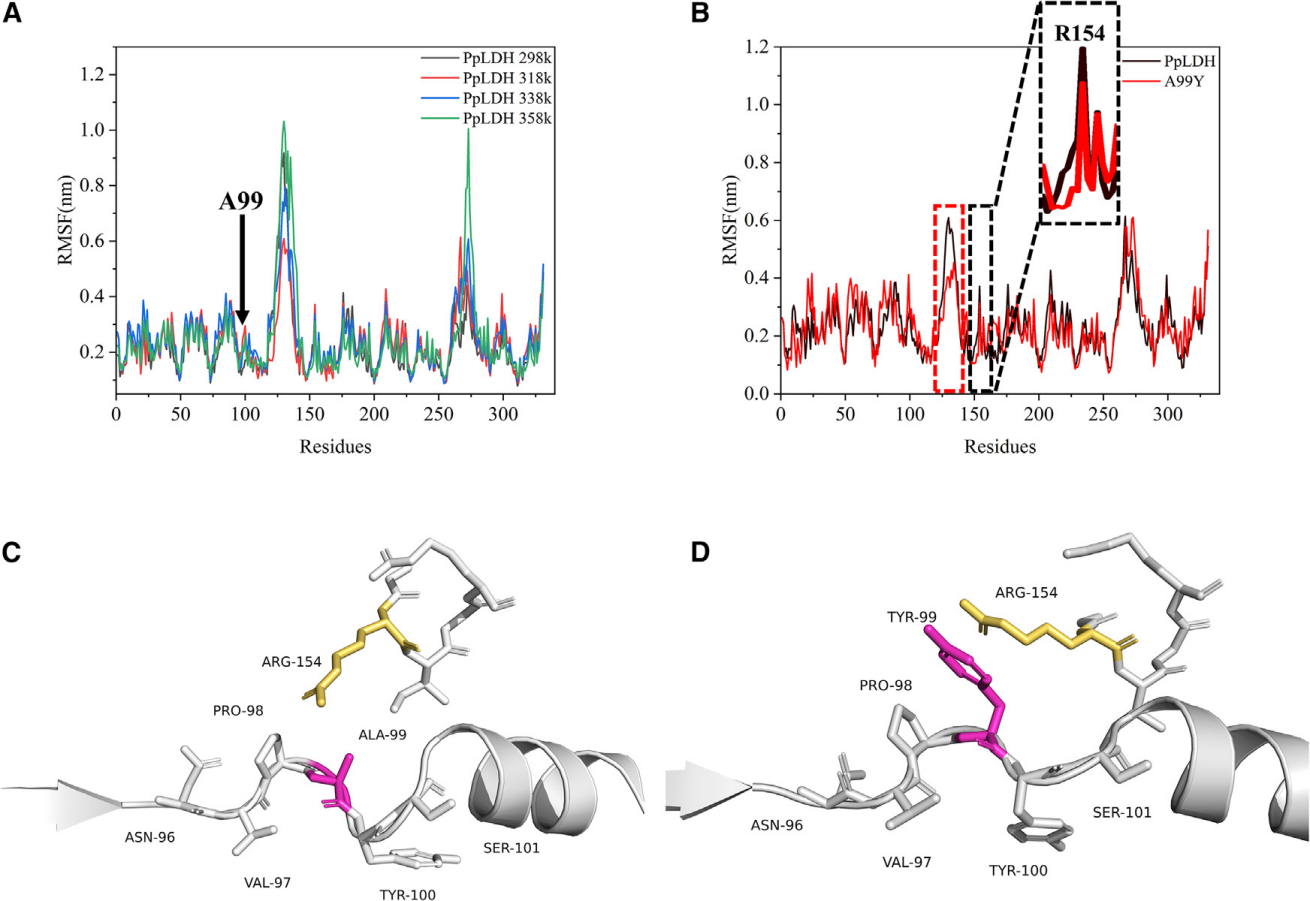
Characteristics of short-loop engineering (A) Comparison of root-mean-square fluctuations (RMSFs) of wild-type PpLDH at 298K (black solid line), 318K (red solid line), 338K (blue solid line), and 358K (green solid line). (B) Comparison of RMSF between wild-type and A99Y mutant at 318K. The red box indicated that A99Y enhanced overall protein stability by reducing flexibility in other structural domains. In the black box, filling the cavity at Ala99 restricted the free movement of Arg154, resulting in decreased RMSF. (C) Interaction diagram between Ala99 and Arg154 in wild type, showing Arg154’s free movement within the cavity. (D) Interaction diagram between Tyr99 and Arg154 in A99Y, illustrating restricted movement of Arg154 due to cavity filling.

Short-loop engineering enhances stability by identifying sensitive residues within rigid regions: throughout the molecular dynamics simulations depicted in Figure 2A, the root-mean-square fluctuation (RMSF) values at the cavity position Ala99 consistently remained below the average, indicating a low B-factor state. The residue’s fluctuations were relatively minimal, suggesting that Ala99 was a rigid site. The strong rigidity of the cavity position arose because it was located within a short-loop region, which typically exhibits lower flexibility compared to longer loop regions. Additionally, the residues flanking the cavity position were hydrophobic, further contributing to its inherent stability. This short-loop region, consisting of six residues, was hydrophobic and exhibited high rigidity, as reflected in the protein’s dynamics simulations, where the RMSF values remained below the average. Consequently, the cavity position, containing this sensitive residue, remained concealed. The B-factor strategy, using molecular dynamics simulations, may have overlooked this position due to its rigidity. However, upon cavity filling, a significant enhancement in protein stability was observed, highlighting the positive effect of this approach. This represents a strategy for enhancing protein stability, distinct from the B-factor strategy. The root-mean-square deviation (RMSD) results corresponding to the aforementioned molecular dynamics simulations process were shown in Figures S1 and S2.

Short-loop engineering improves the rigidity of other structural domains: we conducted mutation and dynamics simulations analyses on the PpLDH Ala99 site, comparing the mutant A99Y model with the wild type. Rather than affecting its own RMSF (rigidity/flexibility), the mutation primarily influenced the overall stability of the 123–137 region (Figure 2B). This finding aligned with the earlier observation that B-factor strategies typically target high RMSF regions for point mutations or saturation mutagenesis to reduce flexibility and enhance overall stability. In contrast, mutations in short loops impacted stability by influencing other regions rather than altering their flexibility, marking another distinctive feature of this strategy.

Impact on the free movement of nearby residues: as shown in Figure 2C, molecular dynamics simulations and protein structural analyses revealed that the wild-type Ala99 site created a cavity, allowing the side chain of residue Arg154 to move freely within this cavity, thereby increasing the flexibility of the region around Arg154. In contrast, Figure 2D showed that substituting Ala with Tyr filled the cavity, restricting the mobility of the Arg154 side chain and shifting it from free movement to constrained movement. This observation was further supported by Figure 2B. This restriction in movement is the key reason for the increase in protein stability.

Un/folding free energy: the un/folding free energy (ΔΔG) is a critical computational metric for evaluating the stability of protein mutations. Using FoldX, we computed the ΔΔG values for the A99F, A99Y, and A99W mutants, as shown in Table 1. FoldX integrates various interactions and entropy changes during folding into a linear energy equation, ΔΔG = ΔG(mutant) − ΔG(wild-type). Negative ΔΔG values indicate a tendency toward increased stability. All the mutants exhibited negative ΔΔG values, confirming that mutations at this position tend to enhance protein stability from the perspective of un/folding free energy. This metric can serve as an evaluative criterion for future short-loop engineering designs.

**Table 1:** The half-life periods and un/folding free energy change of mutants guided by short-loop engineering

Enzyme	Mutants	Half-life period (min)^d^	FoldX-ΔΔG (kcal/mol)
PpLDH^a^	WT	72.6	–
PpLDH	A99F	547.8	−1.02
PpLDH	A99W	469.2	−0.57
PpLDH	A99Y	690	−1.83
UOX^b^	WT	11.1	–
UOX	T75F	17.4	−1.22
UOX	T75W	34.5	−1.47
UOX	T75Y	17.1	−1.54
LDHD^c^	WT	27.4	–
LDHD	E187F	39.2	−0.98

a The heat treatment of PpLDH and its mutants was 45°C.

b The heat treatment of UOX and its mutants was 40°C.

c The heat treatment of LDHD and its mutants was 60°C.

d The changes of residual enzyme activity of all wild types and mutants with different times were shown in Figures S11–S13.

### Enhancing thermal stability of UOX using short-loop engineering

**Figure 3:**
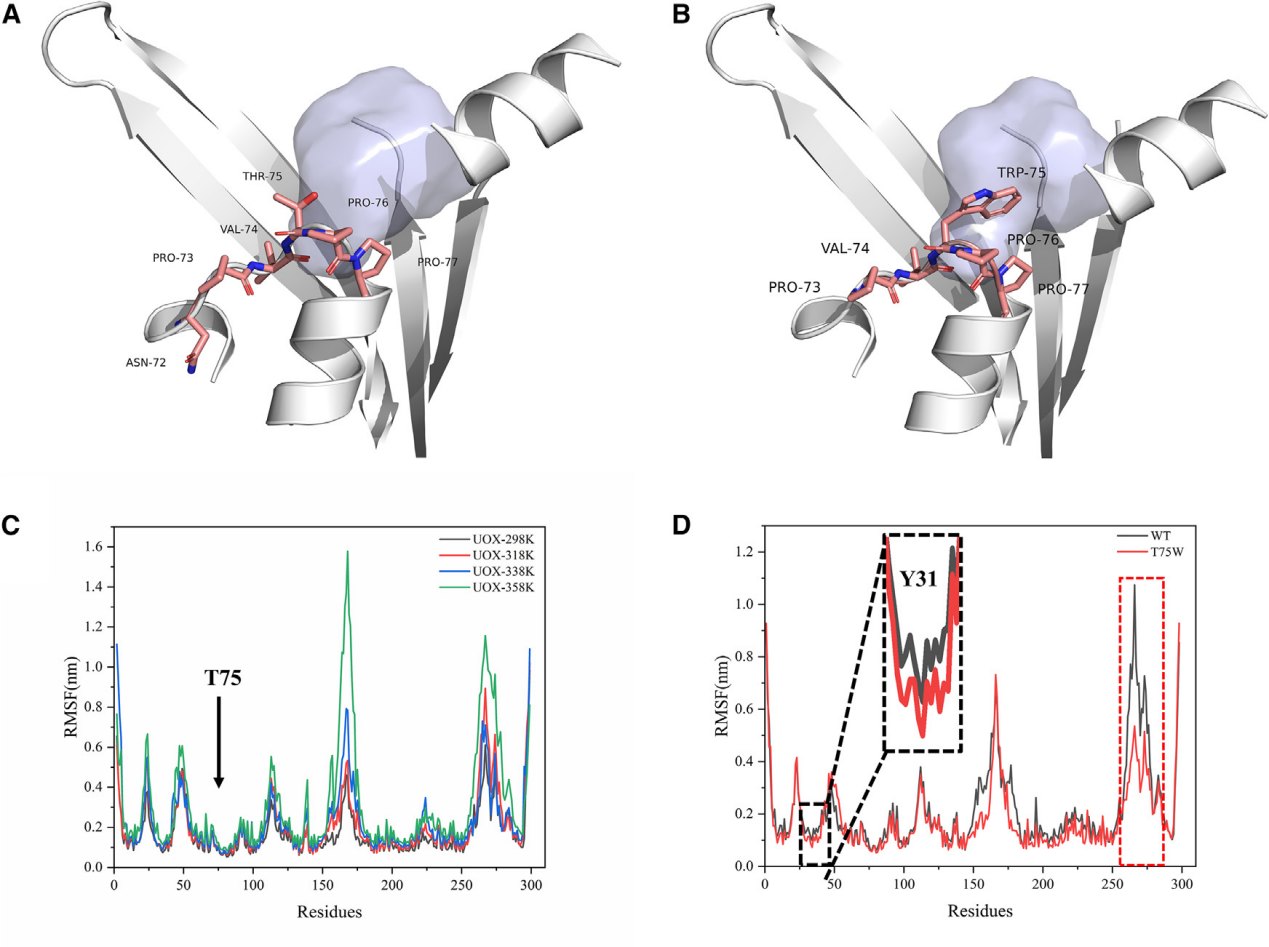
Enhancing thermal stability of urate oxidase using short-loop engineering (A) Wild-type urate oxidase (UOX) exhibited a noticeable cavity at position Thr75 (marked with a solid blue sphere). (B) Partial filling of the cavity at position 75 upon mutation of Thr to Trp. (C) Comparison of root-mean-square fluctuations (RMSFs) of wild-type UOX at 298K (black solid line), 318K (red solid line), 338K (blue solid line), and 358K (green solid line). (D) Comparison of RMSF between wild-type and T75W mutant at 318K. The red box indicated that T75W enhanced overall protein stability by reducing flexibility in other structural domains. In the black box, filling the cavity at Thr75 restricted the free movement of Tyr31, resulting in decreased RMSF.

To validate the universality of the short-loop engineering strategy, we selected UOX from *Aspergillus flavus* as our study subject. Initially, the protein underwent 100 ns of molecular dynamics simulations to stabilize its conformation, followed by the identification of a sensitive residue within the short-loop region. As shown in Figure 3A, residues Asn72, Pro73, Val74, Thr75, Pro76, and Pro77 formed a short loop connecting two α-helical segments. Similar to PpLDH, Thr75 was flanked by hydrophobic residues on both sides. Due to the small side chain of Thr75, it created a cavity with a volume of 884 Å^3^. Aromatic residues Phe, Tyr, and Trp were used to fill this cavity. For instance, the mutation to Trp, as shown in Figure 3B, demonstrated that the mutation partially filled the cavity, reducing its volume to 655 Å^3^.

Furthermore, as shown in Figure 3C, during the molecular dynamics simulations of the wild type at 298K, 328K, 338K, and 358K, the RMSF value of Thr75 consistently remained low, indicating minimal fluctuations and strong rigidity at this site. As demonstrated in Figure 3D, comparative analysis revealed that the mutation at Thr75 affected the overall fluctuations in the 261–277 region, with the RMSF of T75W consistently lower than that of the wild type. This finding aligns with the earlier observation: the cavity at Thr75 in UOX represented a sensitive residue within a rigid segment. Upon cavity filling, the RMSF at 75 remained unchanged, but the overall protein stability was enhanced by reducing flexibility in other structural domains (261–277). The RMSD results corresponding to the aforementioned molecular dynamics simulations process are shown in Figures S3 and S4.

As shown in Figure S5, Tyr31, located near Thr75, exhibited significant fluctuations during molecular dynamics simulations, indicating flexible movement within the cavity. In contrast, the mutation to Trp, as depicted in Figure S6, restricted the movement of Tyr31 due to partial cavity filling, further confirming that short-loop engineering reduces the fluctuations of neighboring residues. This observation was also supported by the RMSF results in Figure 3D, where the flexibility of Tyr31 decreased after the mutation.

We also calculated the un/folding free energy (ΔΔG) for T75W, T75Y, and T75F mutations, as shown in Table 1. All the ΔΔG values were negative, indicating that these mutations enhanced stability from the perspective of ΔΔG. This result aligns with the beneficial effects of short-loop engineering on protein stability in terms of un/folding free energy.

Similar to Ala99 in PpLDH, Thr75 in the UOX short loop (Pro73-Val74-Thr75-Pro76-Pro77) exhibited all the characteristics suitable for short-loop engineering. Consequently, we performed mutations T75W, T75Y, and T75F, followed by *in vitro* expression and stability testing. The wild-type enzyme had a half-life of 11.12 min at 40°C. After short-loop engineering, the stability of uricase at 40°C was significantly enhanced, with T75W showing a half-life 3.1 times longer than the wild type, reaching 34.39 min. This experimental outcome substantiates the effectiveness of short-loop engineering in UOX.

### Enhancing thermal stability of LDHD using short-loop engineering

**Figure 4:**
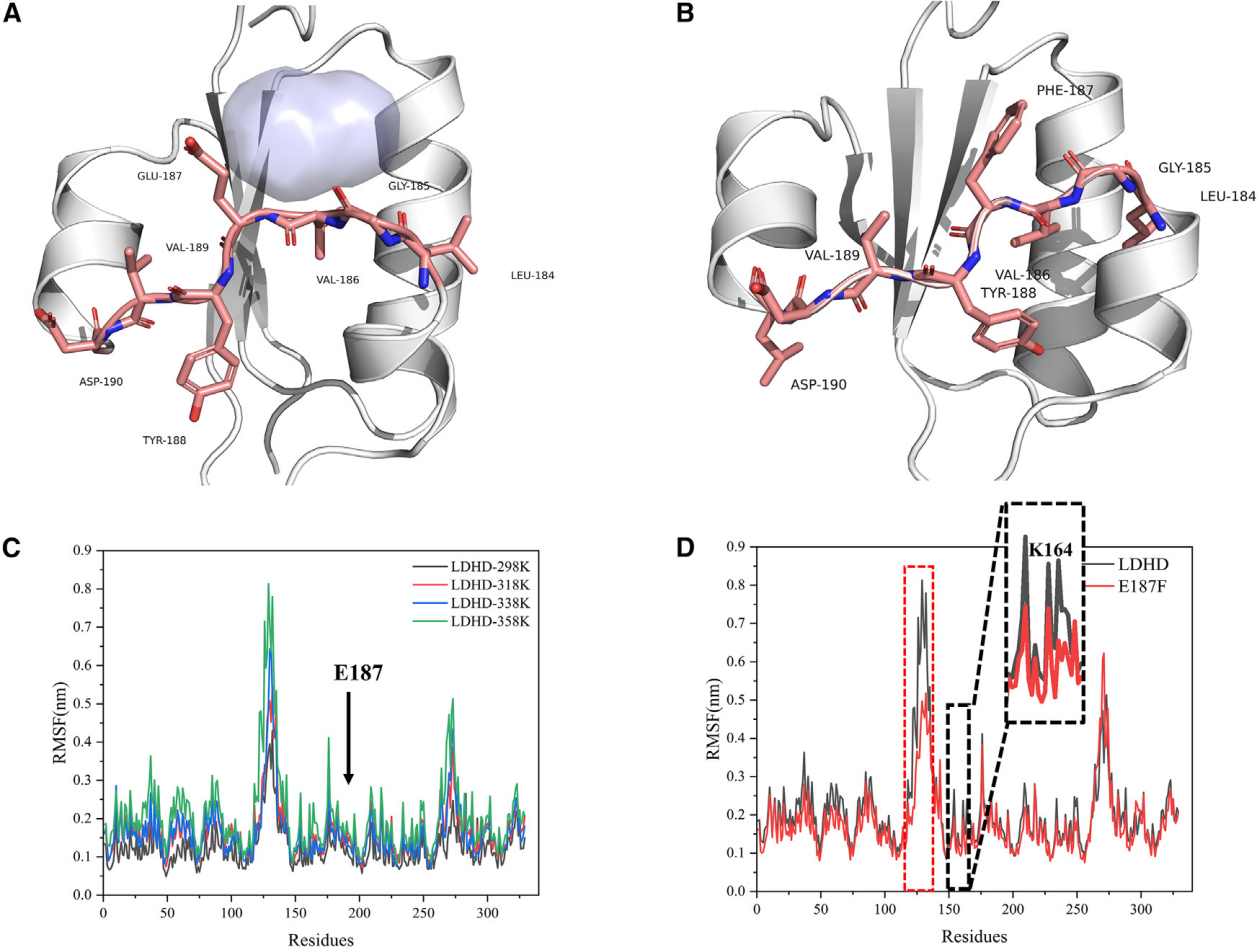
Enhancing thermal stability of D-lactate dehydrogenase using short-loop engineering (A) Wild-type D-lactate dehydrogenase (LDHD) exhibited a noticeable cavity at position Glu187 (marked with a solid blue sphere). (B) Partial filling of the cavity at position 187 upon mutation of Glu to Phe. (C) Comparison of root-mean-square fluctuations (RMSFs) of wild-type LDHD at 298K (black solid line), 318K (red solid line), 338K (blue solid line), and 358K (green solid line). (D) Comparison of RMSF between wild-type and E187F mutant at 358K. The red box indicated that E187F enhanced overall protein stability by reducing flexibility in other structural domains. In the black box, filling the cavity at Glu187 restricted the free movement of Lys164, resulting in decreased RMSF.

To further verify the universality of short-loop engineering, we selected LDHD from *Klebsiella pneumoniae* as our study target. As depicted in Figure 4A, residues Leu184, Gly185, Val186, Glu187, Tyr188, Val189, and Asp190 form a short-loop region connecting two α-helical segments. Similar to PpLDH (Ala99) and UOX (Thr75), Glu187 in LDHD is flanked by continuous hydrophobic residues and forms a cavity with a volume of 150 Å^3^.

We chose the aromatic residue Phe for cavity filling. Results from mutational model construction and molecular dynamics simulations, illustrated in Figure 4B, confirmed successful cavity filling, reducing the cavity volume to below 48 Å^3^.

As shown in Figure 4C, during wild-type molecular dynamics simulations, the RMSF value of Glu187 consistently remained low, indicating strong rigidity at this site, similar to observations in PpLDH and UOX. Additionally, as shown in Figure 4D, comparative analysis revealed that mutation at Glu187 affected the overall fluctuation of the 121–140 region, with the RMSF for the E187F mutation in this region consistently lower than that of the wild type. This observation aligns with earlier findings on cavity filling in PpLDH and UOX: Glu187 represents a sensitive residue concealed within a rigid segment. Upon cavity filling at this sensitive residue position, flexibility in other structural domains was reduced, thereby influencing overall protein rigidity. The RMSD results corresponding to the aforementioned molecular dynamics simulations process are shown in Figures S7 and S8.

Figure S9 shows that Lys164, located near Glu187, exhibited significant fluctuation during molecular dynamics simulations, demonstrating flexible movement within the cavity. In contrast, mutation to Phe, as shown in Figure S10, restricted the movement of Lys164 due to cavity filling, further confirming that short-loop engineering reduced the fluctuation of neighboring residues. This observation was also supported by Figure 4D, where RMSF results showed that the flexibility of Arg164 decreased after the mutation.

According to Table 1, during the LDHD stability redesign process guided by short-loop engineering, the un/folding free energy change of the E187F mutant showed a trend similar to that observed in the previous two enzymes, favoring beneficial alterations. Upon filling the cavity in the short-loop region composed of seven residues, stability was enhanced, with the half-life increasing from 27.4 min to 39.2 min at 60°C. This once again demonstrates the broad applicability of short-loop engineering.

### Standardized process for short-loop engineering

After conducting the aforementioned experiments, it was observed that the proposed short-loop strategy exhibits a certain degree of generalizability. Consequently, a standardized and visualized workflow for this strategy was developed to facilitate the rapid identification of sensitive residues within short loops in proteins. As illustrated further, the process consists of the following steps:

Step 1: Construct an accurate high-resolution protein model using AlphaFold3/AlphaFold2.

Step 2: Visualize the protein structure obtained in the previous step using PyMOL. Hydrophobic residues are highlighted in yellow on the amino acid sequence, and loop regions within the secondary structure are visually represented in red. The three-dimensional structure of the protein is also displayed, with cavities shown as blue spheres using ParkVFinder. This allows for the preliminary identification of sensitive residue positions in the short-loop regions based on characteristics of “cavities in the short-loop region” and “hydrophobic interactions enhance protein stability”.

One of the features of short-loop engineering strategy proposed was the search for sensitive residues. These residues were called sensitive residues because they were located in a rigid region and could not be mined by the B-factor strategy. The rigidity was brought about by two aspects. The first is the short-loop region; the loop region plays a crucial role as an important secondary structure and represents flexibility, and short loops tend to have low flexibility. The second aspect: as mentioned in characteristic of “hydrophobic interactions enhance protein stability” of the paper, there were consecutive hydrophobic residues near the sensitive residues, and stronger hydrophobic interactions better stabilize protein structures. Therefore, we believed that, according to this paper, the short loops used for this strategy were generally composed of 5–7 residues, had consecutive hydrophobic residues, and generated cavities.

Step 3: Perform virtual screening by employing molecular dynamics simulations and calculating the un/folding free energy to assess whether the sensitive residues and large side-chain hydrophobic residue mutants meet characteristics of “short-loop engineering enhances stability by identifying sensitive residues with in rigid regions”, “short-loop engineering improves the rigidity of other structural domains”, “impact on the free movement of nearby residues”, and “un/folding free energy”. If all conditions are satisfied, heterologous expression can be attempted, followed by *in vitro* stability testing for validation.

**Scheme 1:**
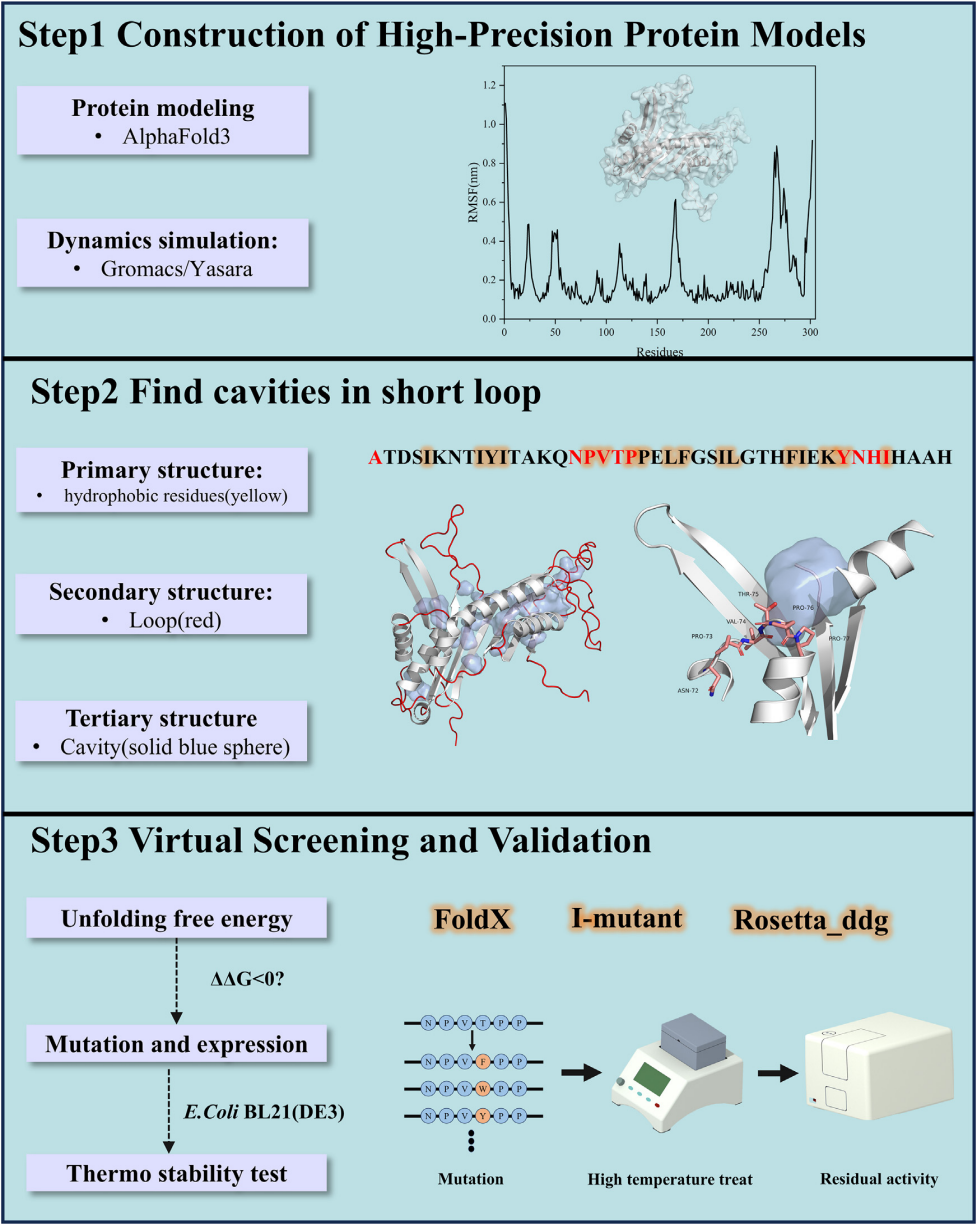
The workflow of the short-loop engineering This is a standardized virtual screening process. Step 1 demonstrates the protein model construction and molecular dynamics simulation process; Step 2 aims to find “sensitive residues” in short loop based on the “cavity finder” visualization plugin; Step 3 is a virtual screening of mutations guided by the calculation of ΔΔG.

In computer-aided design-based protein engineering, computational tools for predicting enzyme thermal stability are expanding, and un/folding free energy calculation can be used to predict the effect of mutations on protein stability, but the accuracy and reliability need to be further improved.^27^ Therefore, computational tools that integrate multiple different algorithms can compensate for the prediction deficiencies of a single algorithm. In Scheme 1, we recommend three tools for un/folding free energy calculation to help us perform virtual screening. This can more accurately obtain mutations with improved stability and reduce the experimental workload.

It is noteworthy that a PyMOL plugin, named “Cavity Finder,” has been developed, and the code is shown in Data S1. This tool automates the second step of the process and enables batch processing of PDB files to locate cavities within short-loop regions.

Both the present strategy and the B-factor strategy require the computation of molecular dynamics to represent the rigid and flexible regions of proteins by RMSF. The difference is that B-factor is guided by the rigidification of the highly flexible region for site finding; the short-loop engineering strategy searches for the sensitive residues on the rigid short loops by structural analysis and molecular dynamics. Therefore, B-factor strategy cannot find the sensitive residues. However, in the molecular dynamics process, it was found that the cavities were filled under the guidance of short-loop engineering; the residues near the sensitive residues were changed from a state where they could flexibly oscillate in the cavity to a restricted state. This was also the reason for the stability improvement. The increase in stability because of the decrease in residue flexibility also complements the idea of the B-factor.

### Conclusion

The loop functions as a “hinge” connecting two structural domains and significantly impacts the dynamics of protein structures. Unlike long loops, short loops are often more rigid and are easily overlooked in strategies aimed at enhancing stability through modifications focused on high flexibility. However, specific sites within rigid domains can also influence protein stability. When continuous hydrophobic segments appear on a short loop and a sensitive residue with a small side chain is present between them, an internal cavity can form, allowing larger movements in adjacent domains. Generally, extensive structural movements are a major factor contributing to instability. In PpLDH, we identified a short-loop region and pinpointed Ala99 as a sensitive residue hidden within a rigid segment, creating a noticeable cavity. Filling the Ala99 site enhanced the stability of PpLDH and reduced the large fluctuations in adjacent domain residues. ΔΔG results also tended to favor stability enhancement. We extended this method to UOX and LDHD, where similar short-loop sensitive residues were identified, and the cavities were filled, resulting in improved stability. We have developed a standardized strategy and a targeted visualization plugin for this method. This approach demonstrates the crucial role of short loops with rigid structures in protein stability. Unlike common strategies that design weakly stable residues in flexible areas to stabilize proteins, this study introduces loop engineering, emphasizing the identification of sensitive residues within specific rigid structures (short loops) to enhance protein thermal stability.

### Limitations of the study

In this study, we found that the stability of the protein was improved after finding sensitive residues on the short loop and filling the cavity. This means that the overall volatility or rigidity of the protein structure was changed. However, when characterizing the dynamics of the protein structure in a solvent environment before and after mutation, the volatility can only be characterized using dynamics simulations. This is a limitation of this study; there are no reported approaches to observe the change of volatility directly after cavity filling.

## Resource availability

### Lead contact

Further information and requests for resources and reagents should be directed to and will be fulfilled by the lead contact, Luo Liu (liuluo@mail.buct.edu.cn).

### Materials availability

All materials generated in this study are available from the lead contact without restriction.

### Data and code availability

• The PpLDH and LDHD protein sequences used in this work were downloaded from NCBI database (www.ncbi.nlm.nih.gov), and the UOX protein sequence and structure were downloaded from PDB database (www.rcsb.org/). All data reported in this article will be shared by the lead contact upon request.
• The original codes in this research have been shown in the Data S1, and the codes have also been uploaded to GitHub (https://github.com/VictorwuwuLLL/CFSL-cavity-finder-in-short-loop).
• Any additional information required to reanalyze the data reported in this article is available from the lead contact upon request.

## Acknowledgments

This work was supported by the 10.13039/501100012166National Key Research and Development Program of China (grant number 2022YFC2104802) and 10.13039/501100001809National Natural Science Foundation of China (grant numbers U21B2098 and 22378015).

## Author contributions

W.Z.: conceptualization, investigation, methodology, experiment, simulation, writing – original draft, and writing – review and editing; Y.L.: simulation and code writing; H.C.: investigation and simulation; L.L.: conceptualization, funding acquisition, supervision, and writing – review and editing; T.T.: conceptualization, supervision, and funding acquisition.

## Declaration of interests

The authors declare no competing interests.

## STAR★Methods

### Key resources table

**Table:** 

REAGENT or RESOURCE	SOURCE	IDENTIFIER
**Bacterial and virus strains**		

*Escherichia coli* TOP10	Weidi	DL1010S
*Escherichia coli* BL21(DE3)	Vazyme	C504-03

**Chemicals, peptides, and recombinant proteins**		

Tryptone	OXODI	LP0042B
Yeast extract	OXOID	LP0021B
Sodium chloride	Macklin	S805257
Kanamucin	Macklin	K963643
IPTG	Solarbio	I8070
Pyruvate	Macklin	P815676
NADPH	Yuanye	S10103
Uric acid	Macklin	U820317
Ni Sepharose 6FF	Solarbio	P2010
Mut Express II Fast Mutagenesis Kit V2 kit	Vazyme	C214
PpLDH (WP_011673315.1)	NCBI	www.ncbi.nlm.nih.gov
LDHD (WP_373647694.1)	NCBI	www.ncbi.nlm.nih.gov
UOX (4d12)	PDB	https://www.rcsb.org/

**Oligonucleotides**		

Primers used for PCR	This study	Table S2

**Recombinant DNA**		

pET-28a (+)	Genewiz	N/A

**Software and algorithms**		

Gromacs	N/A	https://www.gromacs.org/
FoldX	N/A	https://foldxsuite.crg.eu/
Pymol	N/A	https://pymol.org/
Alphafold2	N/A	https://alphafold.com/

**Deposited data**		

Cavity Finder in Short Loop	This study	GitHub: https://github.com/VictorwuwuLLL/CFSL-cavity-finder-in-short-loop and Data S1

### Experimental model and study participant details

In this study, *Escherichia coli* TOP10 cells were used for plasmid amplification, and *Escherichia coli* BL21(DE3) was used for protein expression. The Luria-Bertani (LB) medium (10 g/L tryptone, 5 g/L yeast extract, 10 g/L NaCl) was used for cell growth. The cells were grown in LB medium at 37°C for 12 h. Then they were inoculated into 100 mL shake flasks at a 1% ratio and cultured at 37°C until the OD600nm reached 0.6–0.8. Next, 0.2 mM IPTG (Isopropyl β-D-Thiogalactopyranoside) was added, and the expression was induced for 12 h at 30°C.

### Method details

#### Cloning, expression, and purification

The strategy for constructing the site-saturation method library (SSM) was the NNK method. The NNK strategy means the primers used for PCR are NNK/MNN (N: A/T/G/C; K: G/T; M: A/C), which contain codons encoding all 20 amino acids. By designing primers, multiple bases were added in equal proportions at the mutation site, and the Mut Express II Fast Mutagenesis Kit V2 kit from Vazyme (Nanjing, China) was used for PCR to achieve the construction of the mutant library. Point mutations were also achieved using the same kit, where primers were designed under rational design guidance to complete the mutation process at a single-point location. PCR was performed for 30 cycles consisting of 30 s at 95°C, 15 s at 95°C, 15 s at 60°C, 6 min at 72°C, 5 min at 72°C. Plasmid amplification was completed in *Escherichia coli* TOP10 and ultimately transformed into *Escherichia coli* BL21 for expression.

In this study, approximately 400 single colonies of the above mutants were inoculated into 96 deep-well plates containing 0.6 mL of LB liquid medium and incubated at 37°C for 10 h. Then inoculate into new 96 deep-well plates with 2% inoculum and incubate at 37°C. When the OD600 reached 0.6, 0.2 mM IPTG was added and incubated at 30°C for 12 h. After centrifugation to collect the organisms, 50 mM acetate buffer (pH 5.4) was added for resuspension. Cell fragmentation was accomplished by repeated freeze-thawing three times and the enzyme solution was collected by centrifugation. The enzyme solution was heat-treated at 45°C for 1 h. The heat-treated enzyme activity was determined according to the PpLDH activity assay, with a reaction system of 200 μL.

Both the wild-type and mutant monoclonals of the three enzymes were incubated in LB medium at 37°C for 12 h. Subsequently, they were inoculated into 100 mL shake flasks at a 1% ratio and cultured at 37°C until the OD600nm reached 0.6–0.8. Next, 0.2 mM IPTG was added, and the expression was induced for 12 h at 30°C. The bacterial cells were collected by centrifugation, resuspended, and the cell disruption process was completed using a high-pressure homogenizer to extract the crude enzyme solution. Protein purification was performed using affinity chromatography, where the purification column was pre-equilibrated with lysis buffer. The protein was loaded using a filter membrane, and after washing away impurities with lysis buffer and wash buffer, the target protein was eluted using high-concentration elution buffer to complete the affinity chromatography process. Further purification and desalting were achieved through an ultrafiltration centrifuge tube, ultimately resulting in pure enzymes.

#### Detection of enzyme activity and measurement of thermal stability

The initial enzyme activity of the three enzymes and their mutants was set as 100%, and their thermodynamic stability at a certain temperature was measured, which refers to the change curve of residual enzyme activity over time. The time when the residual enzyme activity is 50% is defined as the half-life of the enzyme at this temperature.

##### PpLDH

50 mM acetate buffer (pH 5.4) was used as the buffer solution, and 10 mM pyruvate and 5 mM NADPH solutions were prepared. The enzyme was incubated at 45°C, and samples were taken at different time points to measure enzyme activity. The reaction system consisted of 1 mL substrate, including 100 μL of pyruvate, 100 μL of NADPH, and 50 μL of enzyme solution. The reaction was carried out at 30°C for 5 min, and 500 μL of anhydrous ethanol was added to terminate the reaction. The specific absorbance at 340 nm was measured using a microplate reader.

##### UOX

Uric acid was dissolved in 50 mM Tris-HCl (pH 8.5) to prepare a 0.1 M uric acid solution. The enzyme was incubated at 40°C, and samples were taken at different time points to measure enzyme activity. The reaction system consisted of 900 μL of substrate, 50 μL of enzyme, and the reaction was terminated with 50 μL of 5 M hydrochloric acid. The reaction was carried out at 30°C for 4 min, and the specific absorbance at 293 nm was measured using a microplate reader.

##### LDHD

The enzyme was incubated at 60°C, and samples were taken at different time points to measure enzyme activity. The remaining methods were consistent with those for PpLDH.

#### Computational methods

All protein model constructions in this article were completed using AlphaFold2(https://colab.research.google.com/github/sokrypton/ColabFold/blob/main/AlphaFold2.ipynb). The MSA (Multiple Sequence Alignment) search was conducted using MMseqs2_uniref_env. The templates used for structure prediction were sourced from the pdb100 database. A total of five structural models were generated and ranked based on the predicted Local Distance Difference Test (pLDDT) scores. Models with the highest scores exceeding 90 were considered for mutant structure analysis.

The molecular dynamic simulations process and result analysis were performed using Gromacs(Version 2021.4). Load the PDB file of wild type and mutants, select the force field: Amber99SB-ILDN, then add ions and perform energy minimization. Pressure pre-equilibrium (NPT) and temperature pre-equilibrium (NVT) were performed, and we can start the simulation. Finally, the Root-Mean-Square Deviation (RMSD) and Root Mean Fluctuation (RMSF) were calculated to indicate the fluctuation of the protein and each residue.

The un/folding free energy calculations (ΔΔG) were obtained using the FoldX plugin in YASARA (Version 21.12.19): the “repair object” was used to optimize protein structure, and the “mutate residue” function was used to perform five parallel calculations to obtain the changes in the un/folding free energy of the mutants. The FoldX energy function includes terms that have been found to be important for protein stability. The free energy of unfolding (ΔG) of a target protein is calculated using the equation: ΔG = Wvdw∗ΔGvdw+WsolvH∗ΔGsolvH+WsolvP∗ΔGsolvP+ΔGwb+ΔGhbond+ΔGel+ΔGKon+Wmc∗T∗ΔSmc+Wsc∗T∗ΔSsc.

Protein structure visualization and graph analysis were completed using PyMOL (Version 2.5.2).

### Quantification and statistical analysis

In the article, the activity of PpLDH and LDHD were measured by detecting the change of NADPH (Abs340nm) over a certain period of time. And the activity of UOX was measured by detecting the change of substrate uric acid (Abs293nm). All the quantitative detection of absorbance was detected by BioTek Synergy H1 Multi-Mode Microplate Reader. And we performed molecular dynamics simulations by Gromacs software to perform statistics for Root-Mean-Square Deviation (RMSD) and Root Mean Fluctuation (RMSF), and the results and figure legends were presented in the manuscript and supporting information. RMSD was used to assess the stability and convergence of the simulation process, data was counted every 10 ps. And RMSF was used to represent the variation of each atom relative to its average position.

## Supplemental information

Document S1. Figures S1–S13 and Tables S1 and S2

Data S1. The code of “Cavity Finder,” related to STAR Methods

## References

[1] Wang Y., Xue P., Cao M., Yu T., Lane S.T., Zhao H. (2021). Directed Evolution: Methodologies and Applications. Chem. Rev..

[2] Li L., Liu X., Bai Y., Yao B., Luo H., Tu T. (2024). High-Throughput Screening Techniques for the Selection of Thermostable Enzymes. J. Agric. Food Chem..

[3] Mesbah N.M. (2022). Industrial Biotechnology Based on Enzymes From Extreme Environments. Front. Bioeng. Biotechnol..

[4] Liu Q., Xun G., Feng Y. (2019). The state-of-the-art strategies of protein engineering for enzyme stabilization. Biotechnol. Adv..

[5] Sanchez-Ruiz J.M. (2010). Protein kinetic stability. Biophys. Chem..

[6] Dill K.A., MacCallum J.L. (2012). The Protein-Folding Problem, 50 Years On. Science.

[7] Nakamura A., Kobayashi N., Koga N., Iino R. (2021). Positive Charge Introduction on the Surface of Thermostabilized PET Hydrolase Facilitates PET Binding and Degradation. ACS Catal..

[8] Rigoldi F., Donini S., Redaelli A., Parisini E., Gautieri A. (2018). Review: Engineering of thermostable enzymes for industrial applications. APL Bioeng..

[9] Bell E.L., Smithson R., Kilbride S., Foster J., Hardy F.J., Ramachandran S., Tedstone A.A., Haigh S.J., Garforth A.A., Day P.J.R. (2022). Directed evolution of an efficient and thermostable PET depolymerase. Nat. Catal..

[10] Markel U., Essani K.D., Besirlioglu V., Schiffels J., Streit W.R., Schwaneberg U. (2020). Advances in ultrahigh-throughput screening for directed enzyme evolution. Chem. Soc. Rev..

[11] Sun Z., Liu Q., Qu G., Feng Y., Reetz M.T. (2019). Utility of B-Factors in Protein Science: Interpreting Rigidity, Flexibility, and Internal Motion and Engineering Thermostability. Chem. Rev..

[12] Li Y., Li C., Huang H., Rao S., Zhang Q., Zhou J., Li J., Du G., Liu S. (2022). Significantly Enhanced Thermostability of Aspergillus niger Xylanaseby Modifying Its Highly Flexible Regions. J. Agric. Food Chem..

[13] Musil M., Jezik A., Horackova J., Borko S., Kabourek P., Damborsky J., Bednar D. (2023). FireProt 2.0: web-based platform for the fully automated design of thermostable proteins. Brief. Bioinform..

[14] Weinstein J.J., Goldenzweig A., Hoch S., Fleishman S.J. (2021). PROSS 2: a new server for the design of stable and highly expressed protein variants. Bioinformatics.

[15] Cui Y., Chen Y., Liu X., Dong S., Tian Y., Qiao Y., Mitra R., Han J., Li C., Han X. (2021). Computational Redesign of a PETase for Plastic Biodegradation under Ambient Condition by the GRAPE Strategy. ACS Catal..

[16] Tu T., Luo H., Meng K., Cheng Y., Ma R., Shi P., Huang H., Bai Y., Wang Y., Zhang L., Yao B. (2015). Improvement in Thermostability of an Achaetomium sp Strain Xz8 Endopolygalacturonase via the Optimization of Charge-Charge Interactions. Appl. Environ. Microbiol..

[17] Kunka A., Marques S.M., Havlasek M., Vasina M., Velatova N., Cengelova L., Kovar D., Damborsky J., Marek M., Bednar D., Prokop Z. (2023). Advancing Enzyme's Stability and Catalytic Efficiency through Synergy of Force-Field Calculations, Evolutionary Analysis, and Machine Learning. ACS Catal..

[18] Wang S., Tang H., Zhao Y., Zuo L. (2022). BayeStab: Predicting effects of mutations on protein stability with uncertainty quantification. Protein Sci..

[19] Sumida K.H., Núñez-Franco R., Kalvet I., Pellock S.J., Wicky B.I.M., Milles L.F., Dauparas J., Wang J., Kipnis Y., Jameson N. (2024). Improving Protein Expression, Stability, and Function with ProteinMPNN. J. Am. Chem. Soc..

[20] Chu H., Tian Z., Hu L., Zhang H., Chang H., Bai J., Liu D., Lu L., Cheng J., Jiang H. (2024). High-Temperature Tolerance Protein Engineering through Deep Evolution. Biodes. Res..

[21] Gu J., Xu Y., Nie Y. (2023). Role of distal sites in enzyme engineering. Biotechnol. Adv..

[22] Nestl B.M., Hauer B. (2014). Engineering of Flexible Loops in Enzymes. ACS Catal..

[23] Boone C.D., Rasi V., Tu C., McKenna R. (2015). Structural and catalytic effects of proline substitution and surface loop deletion in the extended active site of human carbonic anhydrase II. FEBS J..

[24] Li Z., Meng S., Nie K., Schwaneberg U., Davari M.D., Xu H., Ji Y., Liu L. (2022). Flexibility Regulation of Loops Surrounding the Tunnel Entrance in Cytochrome P450 Enhanced Substrate Access Substantially. ACS Catal..

[25] Brinkmann-Chen S., Flock T., Cahn J.K.B., Snow C.D., Brustad E.M., McIntosh J.A., Meinhold P., Zhang L., Arnold F.H. (2013). General approach to reversing ketol-acid reductoisomerase cofactor dependence from NADPH to NADH. Proc. Natl. Acad. Sci. USA.

[26] Guo R., Cang Z., Yao J., Kim M., Deans E., Wei G., Kang S.-g., Hong H. (2020). Structural cavities are critical to balancing stability and activity of a membrane-integral enzyme. Proc. Natl. Acad. Sci. USA.

[27] Bi J., Chen S., Zhao X., Nie Y., Xu Y. (2020). Computation-aided engineering of starch-debranching pullulanase from Bacillus thermoleovorans for enhanced thermostability. Appl. Microbiol. Biotechnol..

