# Supplementary material for: Short-loop engineering strategy for enhancing enzyme thermal stability
Source: iScience. 2025 Mar 11;28(4):112202. doi: 10.1016/j.isci.2025.112202 (PMC11982487; doi:10.1016/j.isci.2025.112202)
Supplement: Document S1. Figures S1–S13 and Tables S1 and S2 [file mmc1.pdf]

iScience, Volume *28*

Supplemental information

Short-loop engineering strategy for enhancing enzyme thermal stability

Wenlong Zhu, Yiheng Liu, Hui Cao, Luo Liu, and Tianwei Tan

Supporting Figures

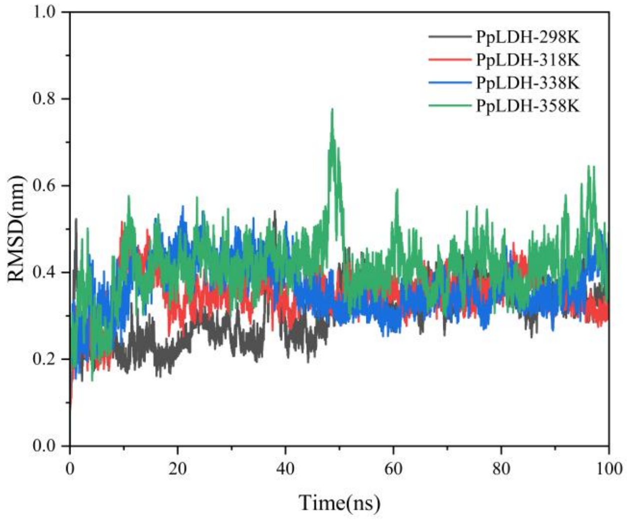

Figure S1. The RMSD of PpLDH at 298K, 318K, 338K and 358K, related to Figure 2. Molecular dynamics simulations of PpLDH at multiple temperatures.

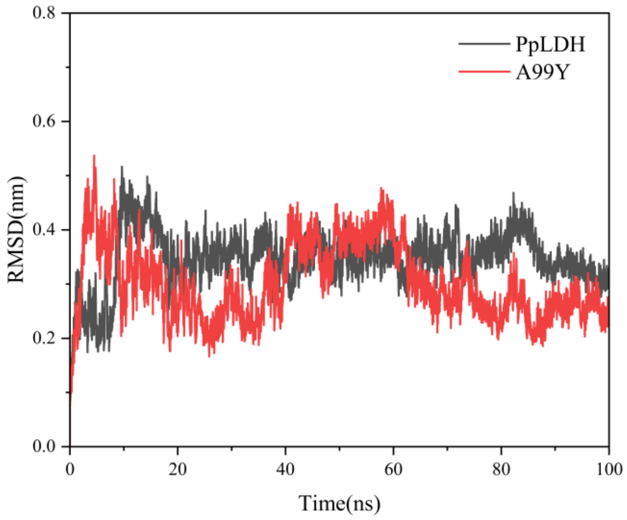

Figure S2. The RMSD of PpLDH and A99Y 318K, related to Figure 2. Molecular dynamics simulations of PpLDH and A99Y at 318K.

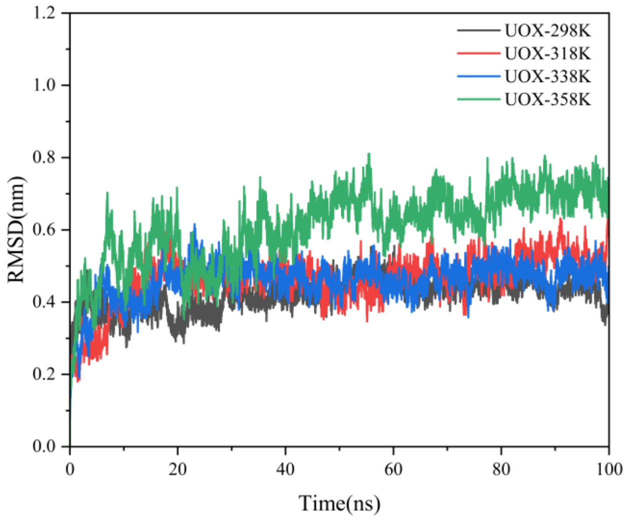

Figure S3. The RMSD of UOX at 298K, 318K, 338K and 358K. related to Figure 3. Molecular dynamics simulations of UOX at multiple temperatures

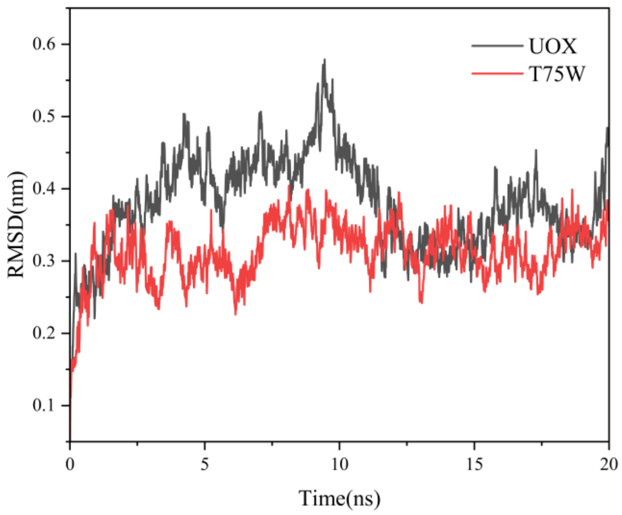

Figure S4. The RMSD of UOX and T75W at 318K, related to Figure 3. Molecular dynamics simulations of UOX and T75W at 318K.

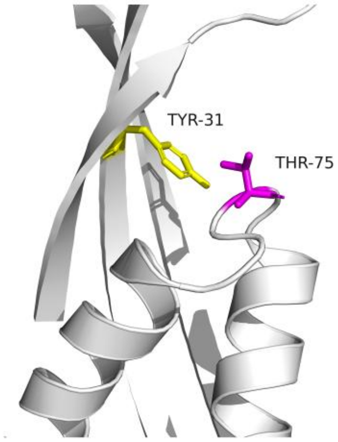

Figure S5. Interaction diagram between Thr75 and Tyr31 in UOX, related to Figure 3. The interaction showed Tyr31's free movement within the cavity.

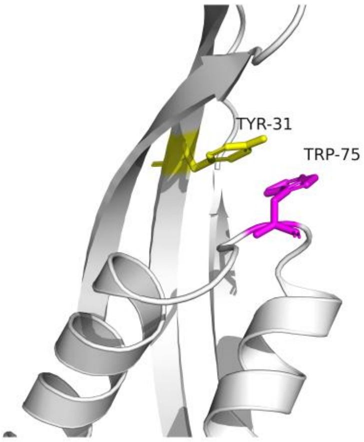

Figure S6. Interaction diagram between Trp75 and Tyr31in T75W, related to Figure 3. The interaction illustrated restricted movement of Tyr31 due to cavity filling.

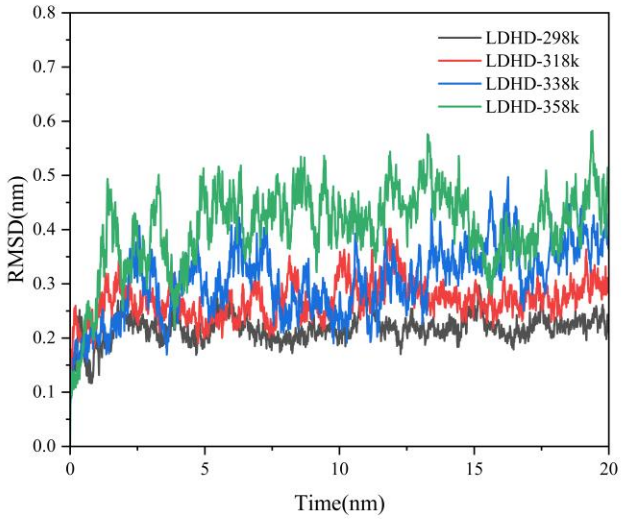

Figure S7. The RMSD of LDHD at 298K, 318K, 338K and 358K, related to Figure 4. Molecular dynamics simulations of LDHD at multiple temperatures.

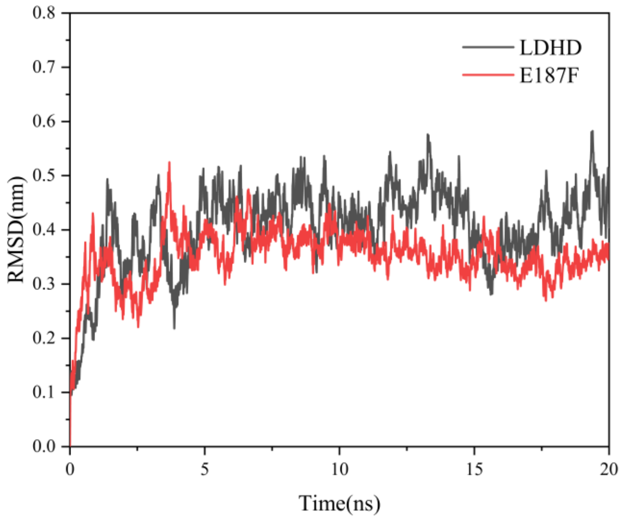

Figure S8. The RMSD of LDHD and E187F at 358K, related to Figure 4. Molecular dynamics simulations of LDHD at 358K.

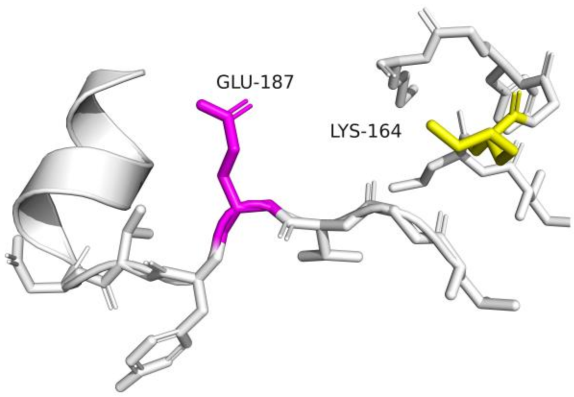

Figure S9. Interaction diagram between Glu187 and Lys164 in LDHD, related to Figure 4.
This interaction showed Lys164's free movement within the cavity.

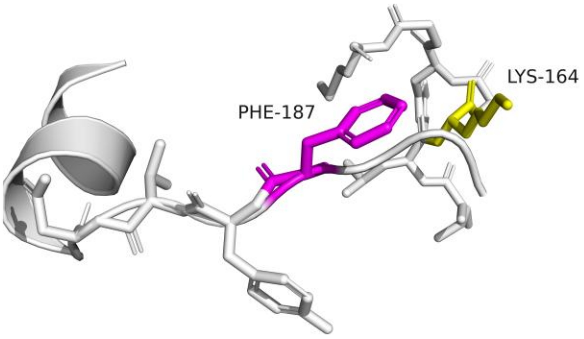

Figure S10. Interaction diagram between Phe187 and Lys164 in E187F, related to Figure 4. This interaction illustrated restricted movement of Lys164 due to cavity filling.

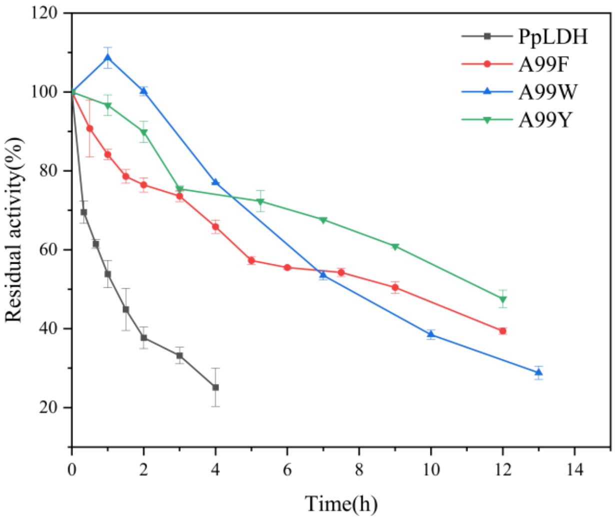

Figure S11. The thermal inactivation curve of PpLDH guided by short loop engineering, related to Table 1. The changes of residual enzyme activity of PpLDH A99F A99W and A99Y with different times at 45℃. Values are shown as mean± s.d. (n = 3 replicates).

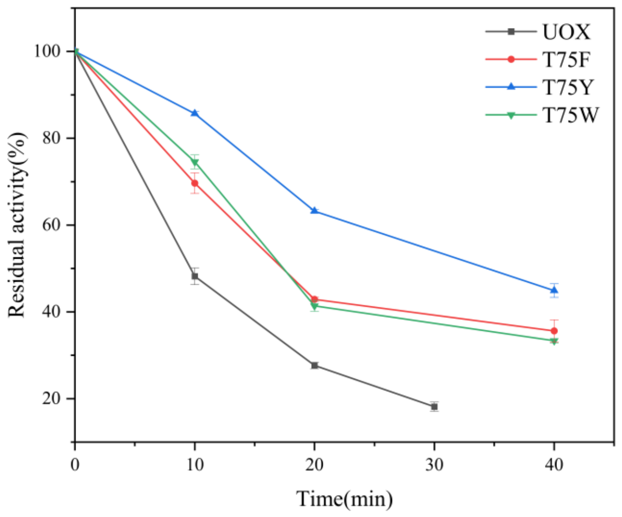

Figure S12. The thermal inactivation curve of UOX guided by short loop engineering, related to Table 1. The changes of residual enzyme activity of UOX T75F T75W and T75Y with different times at 40℃. Values are shown as mean± s.d. (n = 3 replicates).

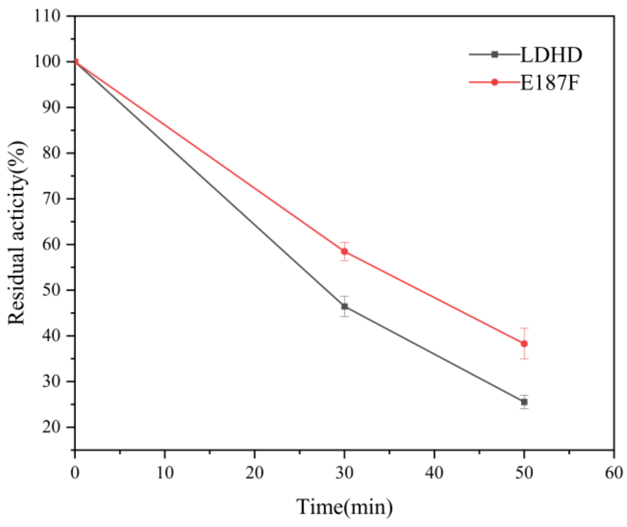

Figure S13. The thermal inactivation curve of LDHD guided by short loop engineering, related to Table 1. The changes of residual enzyme activity of LDHD and E187F with different times at 60℃. Values are shown as mean± s.d. (n = 3 replicates).

Supporting Tables

Table S1 Virtual saturation screening of the Ala99, related to Figure 1.

	ΔΔG(Kcal/mol)					
	N96	V97	P98	A99	Y100	S101
A	0.82	2.36	1.71	-	2.16	0.66
C	0.11	1.93	1.77	-0.66	2.22	-0.64
D	1.91	3.67	1.24	-0.59	2.52	-1.46
E	1.33	4.38	1.02	0.11	1.85	-0.42
F	0.19	11.38	2.03	-0.93	0.38	-1.81
G	0.75	3.4	2.18	1.3	3.13	1.22
H	-0.38	10.11	2.09	-0.02	2.81	0.13
I	-0.56	0.49	1.69	0.04	3.25	2.4
K	-0.13	6.65	1.48	-0.84	2.03	0.22
L	0.9	2.3	1.54	-0.88	2.08	-0.51
M	-1.8	3.27	1.4	-0.28	1.35	-1.67
N	-	3.38	1.65	-0.23	3.06	-0.06
P	0.18	4.13	-	0.66	2.23	10.65
Q	0.65	4.98	1.38	0.1	1.63	-0.57
R	-0.38	11	1.55	-0.30	6.56	0.44
S	1.27	2.97	1.12	-0.73	3.49	-
T	1.45	1.39	2.21	0.11	3.8	3.19
V	-0.46	-	2.02	-0.47	3.05	3.06
W	2.57	14.93	1.74	-0.45	-0.17	1.65
Y	0.16	15.44	1.93	-0.76	-	-1.15

Table S2. Primers used in this study, related to STAR Methods

Primer	Sequences (5'-3')
A99-F	GTGCCGNNKTATAGCCCGATGGCGATTGCGGAACTGAGCGTGA
A99-R	GCTATAMNNCGGCACGTTGGTAATTTTAATGTTGTTGCGTTTCAGC
T75F-F	CCGGTGTTCCCGCCGGAACTGTTTGGCAGCA
T75F-R	CGGCGGGAACACCGGGTTCTGTTTCGCGGTA
T75W-F	CCGGTGTGGCCGCCGGAACTGTTTGGCAGCA
T75W-R	CGGCGGCCACACCGGGTTCTGTTTCGCGG
T75Y-F	CCGGTGTACCCGCCGGAACTGTTTGGCAGCA
T75Y-R	CGGCGGGTACACCGGGTTCTGTTTCGCGGTA
E187F-F	GGGCGTGTTCTATGTGGATCTGGCGACCCTG
E187F-R	CCACATAGAACACGCCCAGTTCCAGCGCG
